# Nuclear Mechanosensation and Mechanotransduction in Vascular Cells

**DOI:** 10.3389/fcell.2022.905927

**Published:** 2022-06-17

**Authors:** Jocelynda Salvador, M. Luisa Iruela-Arispe

**Affiliations:** Department of Cell and Developmental Biology, Feinberg School of Medicine, Northwestern University, Chicago, IL, United States

**Keywords:** cytoskeleton, endothelial, LINC complex, mechanotransduction, nucleus, shear stress (fluid)

## Abstract

Vascular cells are constantly subjected to physical forces associated with the rhythmic activities of the heart, which combined with the individual geometry of vessels further imposes oscillatory, turbulent, or laminar shear stresses on vascular cells. These hemodynamic forces play an important role in regulating the transcriptional program and phenotype of endothelial and smooth muscle cells in different regions of the vascular tree. Within the aorta, the lesser curvature of the arch is characterized by disturbed, oscillatory flow. There, endothelial cells become activated, adopting pro-inflammatory and athero-prone phenotypes. This contrasts the descending aorta where flow is laminar and endothelial cells maintain a quiescent and atheroprotective phenotype. While still unclear, the specific mechanisms involved in mechanosensing flow patterns and their molecular mechanotransduction directly impact the nucleus with consequences to transcriptional and epigenetic states. The linker of nucleoskeleton and cytoskeleton (LINC) protein complex transmits both internal and external forces, including shear stress, through the cytoskeleton to the nucleus. These forces can ultimately lead to changes in nuclear integrity, chromatin organization, and gene expression that significantly impact emergence of pathology such as the high incidence of atherosclerosis in progeria. Therefore, there is strong motivation to understand how endothelial nuclei can sense and respond to physical signals and how abnormal responses to mechanical cues can lead to disease. Here, we review the evidence for a critical role of the nucleus as a mechanosensor and the importance of maintaining nuclear integrity in response to continuous biophysical forces, specifically shear stress, for proper vascular function and stability.

## 1 Introduction

**FIGURE 1:**
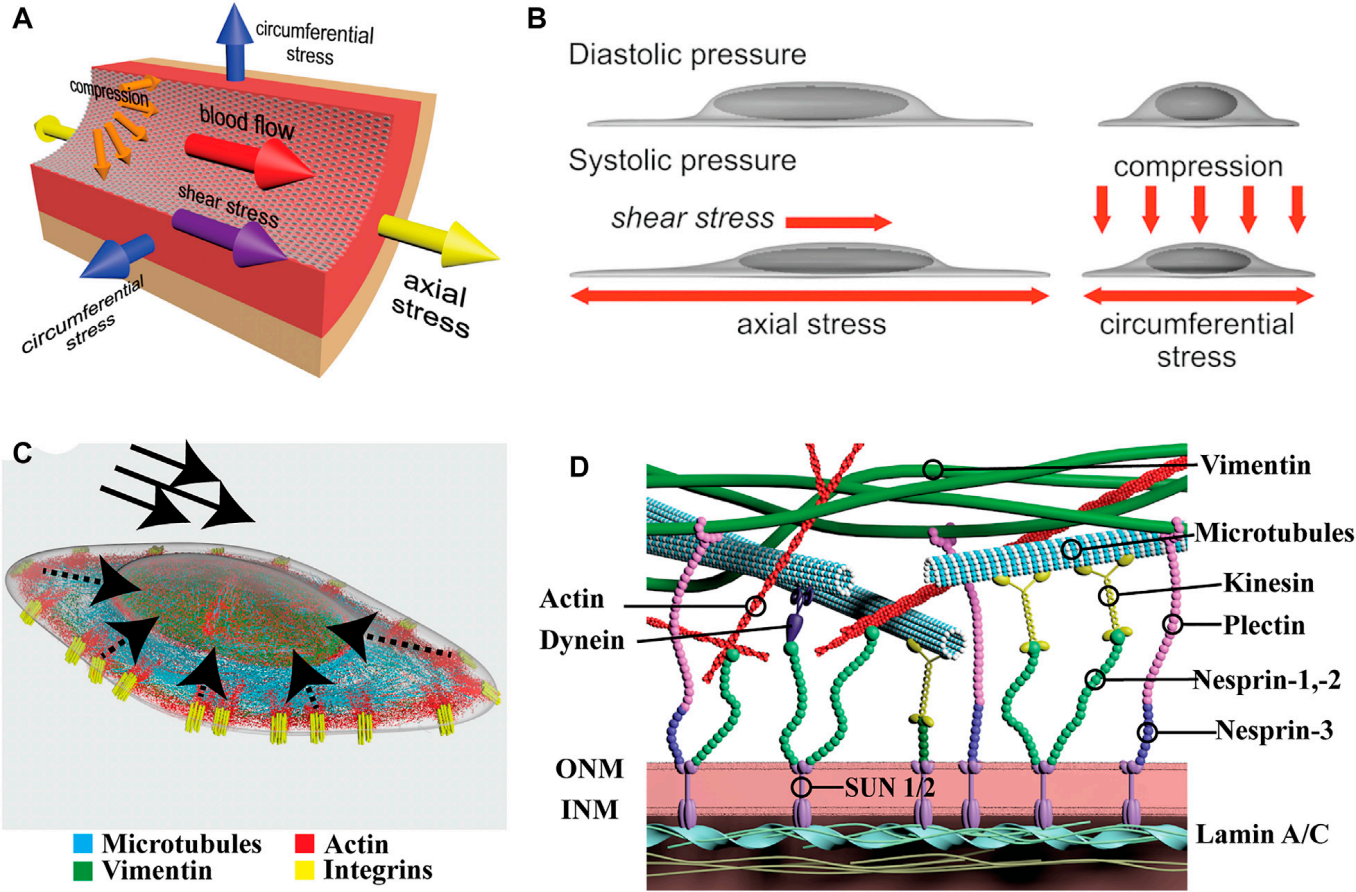
Physical forces in vascular cells and organization of LINC proteins. **(A)** Schematic depicting the major forces present in a large vessel. Arrows indicate the directions of fluid shear stress imposed by flow, and axial, circumferential, and compression stress imposed by distention of the vessel. **(B)** Depiction of nuclear and cytoplasmic changes in endothelial cells under shear stress (left) and compression (right). Note how the nucleus protrudes into the lumen of the vessel and it is directly exposed to flow. **(C)** Organization of the major cytoskeletal filaments in endothelial cells. Actin, in red, connects with integrins, in yellow, within focal adhesions (FAs). Dashed arrows indicate directions of force propagation from FAs to the nucleus. Solid arrows indicate the direction of shear stress. Microtubules (blue) extend throughout the cell and vimentin (green) forms a cage around the nucleus. **(D)** Closer look at the cytoskeletal connections to the LINC complex. The major cytoskeletal filaments connect either directly or indirectly to nesprins. Nesprins anchored in the outer nuclear membrane (ONM) connect to SUN proteins in the perinuclear space. Finally, SUN1/2 proteins at the inner nuclear membrane (INM) connect to the nuclear Lamins which themselves make contact with chromatin.

Large arteries such as the aorta are composed of three main layers: an inner surface of endothelial cells (ECs) in direct contact with circulating blood, followed by vascular smooth muscle cells (vSMCs) in the middle, and connective tissue in the outer surface of the vessel. Due to their location, ECs are directly exposed to shear stress forces imposed by the blood and the cyclic stretch from the pulsatile beating of the heart; while vSMCs and fibroblasts primarily experience the latter **(** Figures 1A,B). Furthermore, depending upon their location, ECs also experience different types of flow. In regions near the heart and at bifurcations, blood flow tends to be *disturbed*, in contrast, in non-branched regions, flow is *laminar*. These distinct types of shear stress significantly alter gene expression with disturbed flow promoting a pro-inflammatory and atheroprone phenotype and laminar flow inducing a non-inflammatory and atheroprotective phenotype (Malek et al., 1999). The realization that physical forces significantly impact gene expression and disease-susceptibility triggered questions as to how the nucleus, and particularly chromatin, recognizes and responds to physical forces associated with vascular physiology.

Endothelial cells sense fluid shear stress *via* several cell surface molecules, including but not limited to ion channels, cell-cell adhesion proteins, and integrins [reviewed elsewhere (Zhou et al., 2014a)]. These, in turn transmit forces and signals to the nucleus through the cytoskeleton (Figure 1C). In fact, when exposed to laminar flow, the nuclei of endothelial cells polarizes downstream flow direction in a matter of hours (Tkachenko et al., 2013). Interestingly, hydrodynamic drag forces *via* air bubbles passing over the endothelial monolayers also resulted in a fast displacement of the nucleus downstream of flow (seconds) showing the rapid response to direct force applied at the cell surface on the nucleus (Tkachenko et al., 2013). Despite the convergence of forces to the nucleus, and demonstation of its direct role as a mechanosensor, the specific molecular cascades initiated and their consequences are only beginning to be elucidated (Caille et al., 2002).

In the last 20 years, evidence for a critical role of the nucleus as a mechanosensing component of the cell was demonstrated in multiple cell types whereby changes in nuclear shape and dynamics in response to biophysical forces directly influenced transcription or compromised chromatin integrity leading to DNA damage (Tkachenko et al., 2013; Cho et al., 2019; Patteson et al., 2019; Xie et al., 2020). For example, mutations in lamins causing Hutchinson-Gilford Progeria Syndrome resulted in severe nuclear dysmorphia in vascular cells leading to chromatin damage, accelerated atherosclerosis and aging (Hennekam, 2006). These findings provided evidence that the nuclei of vascular cells can perceive and mitigate the negative effects of relentless impact of flow and stretch through a series of molecular connections that expand from the cell surface to the nucleus.

The mammalian Linker of Nucleoskeleton and Cytoskeleton (LINC) Complex couples the cytoskeleton proteins to the nucleus *via* proteins that reside within the nuclear envelope (Denis et al., 2021). The LINC complex consists of actin, microtubules, and cytoplasmic intermediate filaments located in the cytoplasm and tethered to the outer nuclear membrane *via* the Klarsicht/ANC-1/Syne-1 homology (KASH) domain proteins, nesprins-1, -2, and -3 (Figure 1D). Nesprins connect to Sad1/UNC-84 (SUN)-domain proteins, SUN1/2, in the inner nuclear membrane which tether to the nuclear lamina composed of the type V intermediate filaments Lamin A/C and Lamin B1/B2, known as lamins. Finally, the lamins interact directly with chromatin at lamina-associated domains (LADs) at the nuclear periphery of the inner nuclear membrane where additional inner nuclear membrane proteins reside. Studies of individual LINC components in EC and vSMCs highlighted their critical roles in nuclear mechanosensing and mechanotransduction (Chancellor et al., 2010; Morgan et al., 2011; Anno et al., 2012; King et al., 2014; Han et al., 2015). Consistent with their predicted contributions, genetic inactivation of several of these LINC components result in vascular defects including embryonic death (Ishimura et al., 2006; Denis et al., 2021). Early evidence of nuclear membrane proteins involvement in vascular development was presented by Ishimura and colleagues. The inner nuclear membrane protein Man1 (also known as LEMD3) represses TGFβ/SMAD signaling by associating with R-Smads (Ishimura et al., 2006). By inserting a Man1-βgeo fusion protein lacking the C-terminal Smad-interacting domain in Man1-null embryos the authors were able to dissect the role of Man1 *in vivo*. Man1 mutant embryos exhibited impaired vascular remodeling with arrest of smooth muscle cell recruitment and deficiency in vascular resilience (Ishimura et al., 2006). Eventually, the primitive vascular plexus is unable to withstand the progressive increase in blood pressure resulting in rupture and death of the embryo due to hemorrhage (Han et al., 2015). Several of such examples have followed providing credence to the concept that nuclear mechanosensing and mechanotransduction is an integral component of normal vascular development and critical to the homeostasis of the circulatory system (Ishimura et al., 2006; Chen et al., 2014; Kim et al., 2018a; Danielsson et al., 2021).

Table 1 summarizes the body of information on the cross talk between the three main cytoskeletal components and LINC proteins for maintaining proper vascular functions in the context of physical forces.

**TABLE 1:** Studies evaluating functions of LINC complex proteins in vascular cells. FSS, fluid shear stress; HUVECs, human umbilical vein endothelial cells; HAECs, human aortic endothelial cells; vSMCs, vascular smooth muscle cells.

LINC protein	Cell type	Forces considered	Consequences of loss and additional observations	References
SUN1/2	Mice: Inducible Sun1fl/fl; Cdh5CreERT2+, Cell: HUVECs	*In vivo* (mice), FSS (cells)	*In vivo* SUN1 depletion leads to reduced radial expansion of developing retinal vasculature, impaired cell-cell junctional integrity. *In vitro* under shear stress, EC SUN1 knockdown (KD) leads to impaired junction stabilization, reduced cortical actin, SUN2-induced increased actomyosin contractility, and impaired perinuclear microtubule network and sprouting angiogenesis.	Buglak et al. (2021)
	Human aortic vSMCs, mouse aortic vSMCs	Static	SUN1/2 KD leads to altered nuclear actin cap organization, reduced cell and nuclear area, increased nuclear/cytoplasmic area ratio, and decreased RhoA activity.	Porter et al. (2020)
Nesprin-1	HUVECs	Normal cyclic stretch	Nesprin-1 KD leads to impaired cell orientation under cyclic stretch, increased traction stress, nuclear height, and focal adhesions, and decreased migration speed in would healing. Actomyosin forces are decreased.	Chancellor et al. (2010)
	HUVECs	Normal cyclic stretch	Nesprin-1 KD leads to decreased nuclear width, increased nuclear strain. No abnormalities in nuclear shape observed.	Anno et al. (2012)
	HUVECs	Static conditions	Nesprin-1,-2 KD leads to increased nuclear area, F-actin stress fibers, impaired localization of Emerin to the inner nuclear membrane, decreased cell migration and 3D network formation in Matrigel assay.	King et al. (2014)
Nesprin-2	Primary rat aortic ECs	FSS	Low shear stress suppressed Nesprin-2 and Lamin A and resulted in increased cell proliferation and apoptosis. Under shear stress, Nesprin-2 regulates transcription factors AP-2 and TFIID. EC-SMC co-culture showed no significant changes in Nesprin2 expression under static or flow.	Han et al. (2015)
	HUVECs	Static conditions	Nesprin-1,-2 KD leads to increased nuclear area, F-actin stress fibers, impaired localization of Emerin to the inner nuclear membrane, decreased cell migration and 3D network formation in Matrigel assay.	King et al. (2014)
Nesprin-3	HAECs	Static and FSS	Nesprin-3 KD induces cell elongation and increased centrosome-nucleus distance under static conditions. Under FSS, Nesprin-3 KD causes impaired centrosome polarization, disrupted plectin and vimentin network organization.	Morgan et al. (2011)
Nesprin-1, -2, -3	HUVECs	FSS	Expression of a DN-KASH construct to displace endogenous nesprins and decouple nucleus-cytoskeleton force transmission leads to impaired EC adhesion, barrier formation, and decreased focal adhesions. Also observed: impaired wound healing and 3D tube formation in a Geltrex assay, decrease in actin stress fibers, and collapse of the vimentin network.	Denis et al. (2021)
	HUVECs	Pulsatile shear stress (PSS), oscillatory shear stress (OSS)	PSS, but not OSS induces increased expression of tight junction proteins ZO-1, Occludin, and lncRNA *MALAT1*. Nesprin-1,-2 KD leads to attenuated expression of these proteins. Additionally, Nesprin-1 nuclear coverage is increased under PSS vs. OSS, and PSS increases the Nesprin-1/SUN2 interaction.	Yang et al. (2020)
Lamin A/C	Rat vSMCs	High cyclic stretch	vSMCs cultured under high cyclic stretch repressed Lamin A/C leading to increased cell proliferation.	Qi et al. (2016)
	Primary rat aortic ECs	FSS	Low shear stress suppressed Nesprin-2 and Lamin A and resulted in increased cell proliferation and apoptosis. Under shear stress, Lamin A regulates activity of STAT1/3/5/6. EC-SMC co-culture showed no significant changes in LaminA expression under static or flow.	Han et al. (2015)
Emerin	vSMCs	High cyclic stretch	vSMCs cultured under high cyclic stretch repressed Emerin expression leading to increased cell proliferation.	Qi et al. (2016)

## 2 The Endothelium as a Mechanosensitive Barrier

As the first layer separating the blood from underlying tissues, ECs constitute a powerful selective barrier that regulates trafficking of molecules and inflammatory cells. Importantly, vascular homeostasis and the execution of barrier functions are tightly integrated with mechanosensation and mechanotransduction. Under static conditions, the endothelium maintains a cobblestone morphology, but once exposed to fluid shear stress, ECs align and elongate in the direction of flow (Barbee et al., 1995; Malek and Izumo, 1996). This change in cell shape is associated with significant alterations in transcriptional profiles that are distinct depending on the magnitude and type of shear stress: laminar or disturbed (including oscillatory and turbulent) experienced by the endothelium.

The growing list of mechanosensing proteins suggests that multiple and concurrent mechanisms are at play to coordinate the sensing of different types of flow and the transduction of downstream responses (Givens and Tzima, 2016). Despite the convergence of these signals at the nucleus, the role of nuclear integrity and chromatin dynamics in facilitating changes in gene expression remain largely unexplored. Also unclear is how gradually reduced levels of shear stress impact the endothelium during the progressive series of bifurcations within the arterial tree. Albeit this is a far more difficult question to address, as it is confounded by the genetic heterogeneity and organotypic influence that alters gene expression of vessels of identical caliber. Meaning, the endothelium of an arteriole in the brain is transcriptionally distinct from the endothelium of the same caliber vessel in the kidney, lung or heart (Augustin and Koh, 2017; Dumas et al., 2021; Gomez-Salinero et al., 2021). Thus, much remains to be uncovered in relation to organ specific read-outs of shear stress at the physiological level.

**FIGURE 2:**
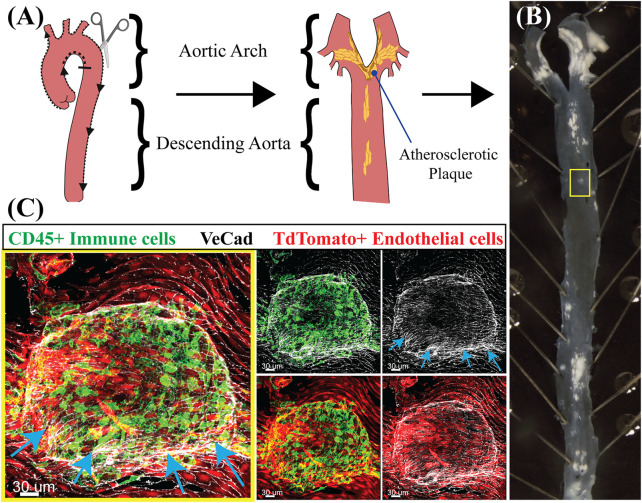
Flow dynamics in atherosclerosis exacerbate the pro-inflammatory state of the endothelium. **(A)** Schematic of an aorta highlighting the aortic arch, where flow is low and oscillatory, and the descending aorta, where flow is high and laminar. After fixation, an aorta is filleted open for en-face staining. The schematic also includes where atherosclerotic plaques (yellow) are expected to be located. Created with BioRender.com. **(B)** Aorta of a mouse injected with AAV-Pcsk9 and fed a high-fat diet for 3 months to induce atherosclerosis. Note the distribution and location of plaques (white) predominantly and at larger sized regions of low, oscillatory flow and branches. The yellow box identifies a small plaque that was subsequently imaged in **(C)**. **(C)** En-face staining of the aorta from **(B)** in the region enclosed by the yellow box. The staining shows an atherosclerotic plaque which is developing under the endothelium raising up the intima layer and a host of inflammatory cells (endothelial cells are Tdtomato +, red, and cell borders marked by VeCad in white). Arrows point to the protruding plaque. Note the large number of CD45^+^ immune cells (green) that are predominantly associated with the endothelial cells covering the plaque.

Atherosclerosis is a prevalent cardiovascular disease of large arteries characterized by the buildup of cholesterol-rich plaques and immune cell infiltration (Figure 2). Atherosclerotic plaques progressively occlude the lumen of the vessel and alter the ability of arteries to regulate blood pressure. Importantly, the incidence of atherosclerosis in the arterial tree is directly correlated with areas exposed to disturbed shear stress which include oscillatory and turbulent flow patterns (Caro, 2009). In these regions (curves and bifurcations), blood flow imposes a chronic pro-inflammatory state demonstrated both *in vitro* and *in vivo* (Nagel et al., 1999; Li et al., 2005; Chiu and Chien, 2011; Davies et al., 2013; Andueza et al., 2020). Genes known to be upregulated in low, oscillatory flow conditions include vascular cell adhesion molecule-1 (*VCAM-1*), intercellular adhesion molecule-1 (*ICAM-1*) and E-selectin (*SELE*), all three involved in the recruitment of inflammatory cells (Nakashima et al., 1998). In addition, these areas of oscillatory flow experience activation of NF-kB, increased ROS production, and upregulation of the chemokine monocyte chemotactic protein-1 (*MCP-1*) (Nakashima et al., 1998). Conversely, in laminar flow regions, the upregulation of the master transcription factors *KLF2* and *4* trigger the activation of anti-inflammatory and anti-thrombotic genes including eNOS (*NOS3*) and thrombomodulin (*THBD*), and the downregulation of pro-inflammatory *VCAM-1* and *MCP-1* (Lin et al., 2005). A summary of the most significant and reproducible transcriptional changes altered by flow is provided in Table 2. For a more in depth characterization of effects of laminar (Diamond et al., 1990; Malek et al., 1994; Ohno et al., 1995; Topper et al., 1996; Topper et al., 1997; Takeshita et al., 2000; Davis et al., 2001; Dai et al., 2004; Chen et al., 2006; Mowbray et al., 2008; Takabe et al., 2011; Fang et al., 2017; Mack et al., 2017; Kim et al., 2018b; Yang et al., 2020; Meng et al., 2022) and disturbed flow (Shyy et al., 1994; Sakai et al., 1997; Chappell et al., 1998; Nakashima et al., 1998; Nagel et al., 1999; Liu et al., 2002; Yu et al., 2002; Sorescu et al., 2003; Platt et al., 2007; Feaver et al., 2008; Civelek et al., 2009; Rhee et al., 2010; Woo et al., 2011; Li et al., 2014; Wang et al., 2016; Ajami et al., 2017; Maurya et al., 2021) on EC transcriptional states we direct readers to excellent reviews (Davies et al., 2013; Jiang et al., 2015; Gimbrone and García-Cardeña, 2016; Simmons et al., 2016; Humphrey and Schwartz, 2021). Finally, loss of proteins involved in mechanosensing and mechanotransduction such as Syndecan-4, ARHGAP18, NOTCH1 and JCAD have been shown to exacerbate atherosclerotic burden (Baeyens et al., 2014; Mack et al., 2017; Lay et al., 2019; Douglas et al., 2020). Combined, these findings reinforce the concept that flow directly impacts the pathophysiological status of the endothelium with direct consequences to vascular health.

**TABLE 2:** List of key endothelial flow responsive genes.

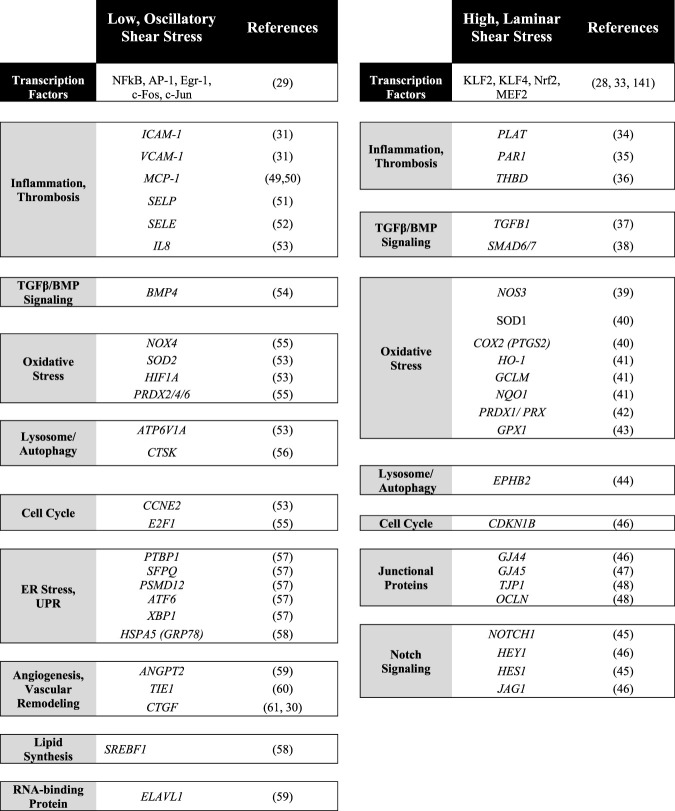

In addition to endothelial cells, mechanotransduction driven by vSMCs is critical for normalizing vascular tone. Vasoactive mediators released by ECs, including nitric oxide and prostacyclin, regulate calcium signaling and acto-myosin contractility in vSMCs (Furchgott and Zawadzki, 1980; Palmer et al., 1987). Additional work suggests that calcium-independent mechanisms of vSMC contraction can be also driven by Rho kinase through the actin cytoskeleton (Numaguchi et al., 1999; Seko et al., 2003; Touyz et al., 2018). Impaired regulation of vSMC contractility and relaxation results in hypertension, which in turns leads to vascular remodeling and altered hemodynamics increasing wall shear stress and strain experienced by EC and vSMCs (Touyz et al., 2018). While at present there is no direct evidence for a direct contribution of nuclear mechanotransduction to hypertension, loss of nuclear envelope proteins alters Rho signaling and acto-myosin contractility in vSMCs providing a possible link between vSMC mechanotransduction and vascular function (Porter et al., 2020).

### 2.1 From the Cell Surface to the Nucleus: Roles of the Cytoskeleton

#### 2.1.1 Actin Microfilaments and Their Contribution to Nuclear Force Sensing

Actin filaments are abundant in ECs and display unique architecture. Thin filaments of cortical actin are present against the membrane and bind to the cytosolic domains of several transmembrane proteins including cadherins (Abu Taha and Schnittler, 2014). In addition, actin stress fibers, which consist of thick bundles of F-actin associated with myosin, directly connect to integrins in focal adhesions. These connections enable the cell to sense forces at the extracellular environment and convey this information to the interior of the cell (Maninová and Vomastek, 2016). Closer to the nucleus, actin organizes into nuclear actin caps with unclear function (Khatau et al., 2009; Chambliss et al., 2013). Importanstly, in response to shear stress, the actin cytoskeleton remodels and adapts to the newly imposed environment (Osborn et al., 2006).

Endothelial responses to shear stress include reinforcement of EC anchorage to the substrate through organized protein clusters known as focal adhesions, this reinforcement relies on actin-integrin connections (Wechezak et al., 1989). At the onset of shear stress, focal adhesions undergo dynamic remodeling due to robust activation of integrins. Expansion of focal adhesions is noted by increase clustering of integrins and recruitment of accessory proteins including, but not limited to talin, paxillin and focal adhesion kinase which expand connections with stress fibers (Jalali et al., 2001). This, in turn, triggers significant changes in the actin cytoskeleton through activation of Rho GTPase (Tzima et al., 2001). Importantly the reorganization of the cytosolic cytoskeleton results in elongation of the cell, a remodeling that reinforces cortical actin and stress fibers along the axis of flow. As cells elongate and align in the direction of shear stress, actin remodeling yields thicker actin filaments at cell-cell junctions, development of apical stress fibers, and reinforced cell-substrate adhesions (Galbraith et al., 1998). Laterally, in regions of cell-cell interactions, actin binds to junctional proteins and contributes to maintain barrier integrity preventing unwanted vascular permeability. At the basal side of the membrane, actin organization in focal adhesions is further remodeled by integrin-mediated signaling to increase strength of adhesion to the underlying basement membrane (Girard and Nerem, 1995).

These robust rearrangements and reinforcements of the actin cytoskeleton are conveyed to the nucleus *via* the LINC complex proteins nesprin-1 and -2, which directly bind to actin. Initially, the assumption was that while sensing the force, nuclear-cytoskeleton connections would not affect actin remodeling in the cytosol, but experimental evidence has revealed otherwise. Genetic inactivation of endothelial nesprin-1 impaired cell alignment, decreased actomyosin tension, and increased nuclear tension in response to cyclic stretching (Chancellor et al., 2010; Anno et al., 2012). Furthermore, under static conditions, nesprin-1 and -2 knockdown resulted in increased actin stress fiber formation (King et al., 2014). More recently, experiments in endothelial cells expressing a dominant negative KASH construct (DN-KASH), which displaces all endogenous nesprins from binding to their cytoskeletal components demonstrated its importance in promoting endothelial cell-adhesion, barrier formation, and focal adhesions (Denis et al., 2021). In support of these results, Yang and colleagues observed a role of nesprin-1,-2 in expression of tight junction proteins ZO-1 and occludin (which bind actin and microtubules), under atheroprotective pulsatile flow (Yang et al., 2020). Expression of these tight junction proteins were attenuated in nesprin-1,-2 knockdown ECs (Meng et al., 2022). An important observation by these authors was the increased degree of nesprin-1 nuclear coverage and nesprin-1/SUN2 interaction under pulsatile but not oscillatory flow highlighting the importance of nucleoskeleton-cytoskeleton connections in endothelial flow responses (Yang et al., 2020). Collectively, these findings reveal an exquisite cross-talk between nuclear tethered actin and the rest of the cytosolic actin cytoskeleton in a manner that impacts global cellular responses to stress. How do nesprin-actin connections regulate cytoplasmic actin network is puzzling, but it uncovers previously unpredicted roles in response to shear stress.

Discrete interactions between nesprin-actin are likely to impact nuclear actin caps, an array of thin actin filamental bundles which regulate nuclear shape *via* the LINC complex (Khatau et al., 2009). A subset of actin filaments from nuclear actin caps was identified to form actin cap-associated focal adhesions (ACAFAs) distinct from cell membrane focal adhesions in that these structures respond more rapidly to substrate stiffness, suggesting a heightened mechanoresponsive function (Kim et al., 2012; Chambliss et al., 2013). Adding to this, Chambliss et al. demonstrated the ability of actin caps to quickly sense and transmit forces using fibroblasts exposed to shear stress. Low shear stress (0.05 dynes/cm^2^) induced actin cap fiber organization as quickly as 30 s after the onset of flow in the absence of alterations in basal stress fibers. Basal stress fiber formation was only observed at a higher level of shear stress (1 dyne/cm^2^), demonstrating rapid adaptation to low shear stresses (Chambliss et al., 2013). Cessation of flow led to the eventual disappearance of actin caps more quickly than basal stress fiber disappearance, further supporting the concept that actin caps are particularly flow responsive. To investigate the mechanism by which these nuclear actin caps formed in response to shear stress, the authors inactivated nesprin-2G (binds actin) and nesprin-3 (binds vimentin intermediate filaments). Cells lacking these nesprins prevented the formation of shear-dependent actin caps, with loss of nesprin-3 displaying a greater impairment in nuclear actin cap formation (Chen et al., 2014).

Importantly, actin is also present inside the nucleus with important nuclear-specific functions. Nuclear actin facilitates repair of DNA double-stranded breaks, maintains nuclear structure in response to replication stress, and regulates chromatin mobility (Belin et al., 2015; Lamm et al., 2020; Scheffler et al., 2022). In addition, nuclear actin and actin-related proteins (ARPs) have been previously identified as components of chromatin remodeling complexes including SWI/SNF (BAF) (Wada et al., 1998; Rando et al., 2002; Szerlong et al., 2008; Kapoor and Shen, 2014). More recent findings described in a preprint by Moonen and colleagues demonstrated that the shear stress induced EC transcription factor KLF2 interacts with the SWI/SNF chromatin remodeling complex (Moonen et al., 2020). This allows chromatin remodeling of atheroprotective genes to become more accessible. It is therefore likely that in vascular cells nuclear actin is a constituent of this chromatin remodeling complex that is sensitive and responsive to shear stress.

#### 2.1.2 Microtubules and Nuclear Responses to Physical Forces

Microtubules, known primarily for their role in protein transport and chromosome segregation in mitosis also play important roles in vascular cell mechano-responses. In 1996, Malek and Izumo demonstrated the requirement of a dynamic microtubule network for EC alignment in response to shear stress (Malek and Izumo, 1996). Microtubules in ECs are also responsible for maintaining cell polarity in response to fluid shear stress. As ECs elongate in the direction of flow the microtubule organizing center moves downstream of the nucleus in the direction of flow (Tzima et al., 2003). Shear stress induced planar cell polarity in endothelial cells also display microtubule stabilization indicated by increased acetylation, a modification that conveys mechanical resilience (McCue et al., 2006; Portran et al., 2017; Xu et al., 2017). Whether other post-translationally modified microtubule populations interact with the LINC complex has not been studied.

Microtubules connect to the nucleus through the interactions of kinesin/dynein to nesprins (Figure 1D). Studies using centrifugal forces in fibroblasts demonstrated the specific role of microtubules in nuclear localization (Zhu et al., 2017). Nuclear movement on microtubules occurs through dynein binding to nesprin-2G and SUN1. However, when the nucleus was displaced towards the front of the cell, nuclear repositioning was driven by actin *via* nesprin-2G and SUN2 (Zhu et al., 2017). In cells where nuclear positioning is important for cell function, including in neurons, skeletal muscle, and hair follicle cells, nuclear movement is driven by a similar process using microtubule motor proteins and nesprin interactions (Wilson and Holzbaur, 2015; Holt et al., 2019; Gonçalves et al., 2020; Taiber et al., 2021). Studies of nesprin inactivation in ECs have focused on actin-specific consequences (described previously), while effects on microtubule cytoskeleton and dynamics being less clear. Nonetheless, the identification of unique microtubule-motor protein-nesprin-SUN interactions suggest cell type specific functions in response to shear stress that are yet to be elucidated.

#### 2.1.3 Intermediate Filament Vimentin and Nuclear Resilience

Vimentin is a type III intermediate filament unique among the cytoskeletal filaments due to its high viscoelastic properties that make vimentin resistant to breakage at high strains (Janmey et al., 1991). In early experiments using actin, microtubules, and vimentin polymers, Janmey and colleagues demonstrated that among the three major cytoskeletal proteins, microtubules are most deformable, but break when subjected to high strains. Actin is the least deformable breaking when subjected to low stains. In contrast, vimentin is deformable at low strains, but retains its elasticity at high strains resisting breakage (Janmey et al., 1991). These findings supported vimentin’s unique contributions in maintaining cell integrity. Indeed, while the protective mechanical role of intermediate filaments in epithelial cells (keratins) is well understood, a similar role in vascular cells is less clear (Jones et al., 1998; Wang et al., 2020). This is remarkably surprising given that vimentin is abundantly expressed by both EC and vSMCs and its expression is high in vascular beds that experience high levels of shear stress (Schnittler et al., 1998). Given its abundance and mechanical properties, it has been difficult to reconcile that vimentin-null mice are viable, fertile and disease-free (Colucci-Guyon et al., 1994). Nonetheless, further evaluation has uncovered a number of defects in vimentin null mice in a large number of tissues and pathological conditions (Patteson et al., 2020; Ridge et al., 2022), further supporting the mechanical and non-mechanical roles of this intermediate filament (Dai et al., 2004).

The first studies to evaluate the functional consequences of vimentin loss on vascular function were performed on carotids and mesenteric resistance arteries from vimentin-null mice. While mesenteric resistance arteries of vimentin null mice exhibit no significant structural changes nor alterations in myogenic tone, they showed impaired flow-induced dilation, demonstrating a requirement for vimentin in response of cells to flow forces (Schiffers et al., 2000). Furthermore, Schiffers and colleagues showed that flow-induced remodeling of carotid arteries was altered in vimentin-null mice (Schiffers et al., 2000) and a separate study confirmed impaired contraction and relaxation in thoracic aortae (Langlois et al., 2017). These studies motivated future investigations on vimentin in vascular cells.

Vimentin was shown to be necessary for initiation of angiogenesis *in vitro*, however such models are primarily driven by growth factors and in the absence of shear stress (Kwak et al., 2012). More recently, vimentin was implicated in the regulation of Notch signaling in ECs (Antfolk et al., 2017; van Engeland et al., 2019; Salvador et al., 2022). This is particularly relevant as Notch signaling is essential for vascular development and mechanotransduction (Mack et al., 2017). A positive correlation between vimentin and expression of the Notch ligand Jagged1 was first identified by Antfolk and colleagues (Antfolk et al., 2017). Analysis of mouse embryonic fibroblasts confirmed interactions between vimentin and Jagged1 and showed that absence of vimentin impaired Notch signaling (Antfolk et al., 2017). The consequences of vimentin loss were also confirmed *in vivo* where vimentin knock-out embryos displayed reduced vascularization supporting a role for vimentin in regulating Jag1 (proangiogenic) versus Dll1 (anti-angiogenic) signaling in vasculature (Antfolk et al., 2017). Subsequent studies from this group demonstrated that under shear stress conditions, vimentin phosphorylation at Serine38 was necessary for Notch transactivation in endothelial cells (van Engeland et al., 2019).

Integrin-vimentin interactions were noted at focal adhesions in ECs which are likely to impact shear-stress responses. However, further studies into these vimentin-associated matrix adhesions are needed to understand the functional relevance of vimentin at those sites (Gonzales et al., 2001; Tsuruta and Jones, 2003).

At the nucleus, the LINC complex interacts with vimentin exclusively through nesprin-3, *via* plectin (Ketema and Sonnenberg, 2011). Patteson and colleagues described the organization of vimentin cage-like structures surrounding the nucleus in mouse embryonic fibroblasts. These vimentin cages serve to maintain nuclear integrity in cells migrating across tight spaces and loss of vimentin resulted in DNA-damage following nuclear constriction (Patteson et al., 2019). In addition, DN-KASH expressing endothelial cells also display collapse of the vimentin network suggesting vimentin’s close association with the nucleus in ECs likely plays a similar protective role (Denis et al., 2021). Studies on ECs *in vitro* using nesprin-3 knockdown surprisingly promoted an elongated cell phenotype under static culture conditions and an increase in centrosome to nucleus distance (Morgan et al., 2011). When cultured under shear stress, nesprin-3 knockdown ECs displayed impaired centrosome polarization, and a disrupted plectin and vimentin network organization.

Together, these findings have cemented vimentin’s role in vascular function, though the complex nature of vimentin regulation (phosphorylation) and range of functions in cells make studying vimentin’s role in mechanosensing and mechanotransduction a challenging process.

## 3 Functional Interactions Between Cytoskeletal Components and Their Collective Contribution to Dissipate and Convey Forces to the Nucleus

Due to their interconnections, the unique functional properties of actin, microtubules, and intermediate filaments relay information to the nucleus in unison and also partake to dissipate the potentially drastic impact of physical forces (Wang et al., 2009; Ingber et al., 2014). Mathematical predictions coupled with experimental data have clarified how force dissipation occurs throughout the cell *via* actin, microtubules and under certain conditions also vimentin (Wang and Suo, 2005; Na et al., 2008; Block et al., 2018; Hu et al., 2019). Furthermore, connections between distinct cytoskeletal components have been shown to partially compensate when deficiencies occur. For example, Hookway and colleagues showed that organization of vimentin’s dynamic network (specifically transport and reorganization of vimentin) is driven by microtubules, but was not altered when microtubule dynamics was impaired (Hookway et al., 2015). In contrast, in migrating cells, it is vimentin that acts to stabilize microtubules directing cell migration (Gan et al., 2016; Schaedel et al., 2021). These studies brought to light how microtubules and vimentin filaments complement one another and might compensate for individual deficiencies.

Interactions between vimentin and actin are known to be important for mitosis. During mitosis cortical actin and vimentin interactions increase, and filaments appear to be interwoven, allowing for proper progression of mitosis (Duarte et al., 2019; Serres et al., 2020). A recent study by Wu and colleagues characterized the interactive nature of vimentin-actin connections in detail (Wu et al., 2022). Analysis of vimentin-actin networks located at the cell cortex of mouse embryonic fibroblasts revealed the presence of vimentin in actin stress fiber bundles and further demonstrated that together they are responsible for cell resilience (Wu et al., 2022). In relation to cytoskeletal-nucleus connections, microtubules and desmin intermediate filaments were shown to control nuclear shape and integrity in cardiomyocytes. Specifically, depletion of desmin leads to abnormal nuclear shape that is driven by microtubules (Heffler et al., 2020). How impairing this cytoskeletal cross-talk alters mechanosensing and mechanotransduction in ECs and SMCs will be an important complement to the above studies on LINC-dependent mechanotransduction.

## 4 THE LINKER OF NUCLEOSKELETON AND CYTOSKELETON COMPLEX IN VASCULAR SMOOTH MUSCLE AND FIBROBLASTS

Vascular smooth muscle cells (vSMCs) and fibroblasts experience cyclic stretch due to the continuous pumping of the heart and rely on their LINC complexes to regulate proliferative, migratory, and contractile functions. Using vSMCs cultured under high cyclic stretch, Qi and colleagues demonstrated that lamin A/C and emerin, both inner nuclear membrane proteins, impact cell proliferation (Qi et al., 2016). High cyclic stretch reduced lamin A/C and emerin levels leading to increased cell proliferation both *in vitro* and *in vivo*. Reduction of lamin and emerin levels under static conditions also increased proliferation (Qi et al., 2016). Supporting these findings, overexpression of lamin and emerin reduced proliferation. Interestingly, another LINC protein SUN1/2, appears to regulate the actin cytoskeleton that covers the nucleus. Under static conditions SUN1/2 knockdown in vSMCs leads to altered nuclear actin cap organization, reduced cell and nuclear area, and decreased RhoA activity (actomyosin activity) (Porter et al., 2020). Collectively, these studies highlighted the unexpected contribution of LINC proteins in cytoskeletal-dynamics, migration and proliferation.

LINC complex proteins are also involved in the DNA damage response in vascular cells. Lamin A anchors DNA damage repair foci and nesprin-2 functions as a scaffold for ERK-mediated ATM/ATR signaling following DNA damage and double strand DNA repair (Liu et al., 2013; Warren et al., 2015). These studies also highlighted that accumulation of pre-lamin A, the immature form of lamin A, in vSMCs result in chronic DNA damage signaling and lead to accelerated vSCM aging (Liu et al., 2013). Surprisingly, loss of lamin A did not impair double stranded DNA repair, in contrast to nesprin-2 depletion which attenuated downstream ATM/ATR signaling and also lead to increased chromatin instability (micronuclei formation) (Warren et al., 2015). In fibroblasts, SUN proteins were observed to interact with components of the DNAPK complex involved in double stranded DNA repair (Lei et al., 2012). Fibroblasts lacking SUN1/2 display increased cell cycle arrest and impaired double strand DNA repair due to lack of SUN1/2-DNAPK complex formation through unclear mechanisms. Thus, LINC complex proteins are *de facto* involved in DNA damage responses.

## 5 Modulation of Epigenetic and Chromatin States by Physical Forces

Despite the well characterized role of integrin-mediated signaling in mechanotransduction, the direct consequences of force transmission from focal adhesions to the nucleus are far more robust than anticipated (Maniotis et al., 1997; Madrazo et al., 2017). Using Chinese hamster ovary cells, Tajik and colleagues demonstrated that applied force to a cell *via* integrins attached to a magnetic bead led to chromatin stretching and such stretching resulted in rapid transcriptional changes (Tajik et al., 2016). Both stretching and transcriptional changes were dependent on stress amplitude and the stretch-mediated transcription was driven by actomyosin contraction. This work provided definitive proof that force applied to integrins can effectively be transmitted to the nucleus leading to changes in chromatin state and also resulting in transcriptional alterations. The findings reinforced pursuit of lingering questions in the field of vascular biology: Do epigenetic modifications alter the ability of chromatin to sense force? Does force alter the 3D-organization of chromatin in the nucleus? Is the organization of chromatin in the nucleus (peripheral/hetero vs. central/euchromatin) sensing forces differently? And, are there specific transcription factors able to sense physical forces and trigger a larger coordinated response?

During the last two decades, two transcription factors have emerged as precise sensors of shear stress: KLF2 and KLF4 (Parmar et al., 2006; Hamik et al., 2007). Both are rapidly and strongly induced by shear stress and regulate a cohort of mechanoresponsive genes. Laminar flow promotes KLF2/4 atheroprotective activity, while disturbed flow decreases KLF2/4 activity resulting in a pro-inflammatory endothelial status (Dekker et al., 2002; Parmar et al., 2006; Hamik et al., 2007; Dunn et al., 2015). Jiang and colleagues analyzed the epigenetic outcomes of endothelial cells cultured under pulsatile undisturbed flow versus oscillatory disturbed flow in an effort to highlight changes in ECs in regions more susceptible to atherosclerosis (Jiang et al., 2014). Analysis of the methylation profile of KLF4 uncovered remarkable plasticity whereby promoter hypermethylation was significantly increased in disturbed flow conditions reducing KLF4’s ability to regulate pro-inflammatory targets. Work from other groups complemented these studies demonstrating that shear stress changed the methylation patterns of endothelial transcription factors by modulating the abundance of DNA methyltransferases (Zhou et al., 2014b; Dunn et al., 2014).

The impact of epigenetics on both sensing and responding to physical forces has been the subject of continuous effort first studied by Illi and colleagues almost 20 years ago. Their work provided proof that shear stress induces post-translational modifications on histones (Illi et al., 2003). Developments in next-generation sequencing technologies and its application to chromatin biology facilitated studies of flow-induced changes at the single cell level. Using a combination of single-cell RNA sequencing and scATAC-seq, Andueza and colleagues demonstrated shear stress-dependent changes in endothelial chromatin accessibility. By employing a partial carotid ligation model to create disturbed flow in a region normally experiencing laminar flow, the authors found that EC transcriptional program was significantly altered in response to disturbed flow in a time-dependent manner (Andueza et al., 2020). Cells experiencing the newly induced disturbed flow changed their atheroprotective gene signature to an inflammatory gene signature [as per upregulation of pro-atherogenic CTGF (Oemar et al., 1997; Andueza et al., 2020)]. Under longer periods of exposure to disturbed flow, the endothelium became more heterogenous with some of these cells developing EndoMT (endothelial-mesenchymal transition) transcriptional signatures, shown by the increased chromatin accessibility and transcript levels of genes normally restricted to vSMCs and fibroblasts. Recent work by Tsaryk and colleagues supported previous findings of changed chromatin states in response to different shear stress, but added the interesting finding that endothelial enhancers switch from ETV/ETS to KLF4 binding sites (Tsaryk et al., 2022). ETV/ETS is an endothelial transcription factor critical to differentiation and organization of vascular cells early during development but it is subsequently repressed to allow vascular development to proceed normally (Hayashi et al., 2012).

Following these discoveries at the epigenetic level, studies of chromatin biology have provided evidence that nuclear shape and nuclear envelope integrity can directly influence chromatin and gene expression. Several studies have demonstrated that both externally applied and cell-generated cytoskeletal forces compressing the nucleus can lead to nuclear envelope rupture and chromatin damage [reviewed in (Shah et al., 2017)]. This evident in pathological conditions, like progeria, where mutations in lamin result in nuclear abnormalities in vSMCs that impact chromatin and gene expression (Kim et al., 2021). The nuclear cytoskeleton, and particularly lamins are critical in protecting protect chromatin against physical forces.

## 6 Lamins: The Nuclear Intermediate Filaments That Interact With Chromatin

The A-type and B-type lamins are type V intermediate filaments that provide mechanical resilience to the nucleus (Lammerding et al., 2006). Through their interactions with heterochromatin at lamin-associated domains (LADs), they strongly contribute to the organization of chromatin in the nucleus (Dechat et al., 2008; McCord et al., 2013). Heterochromatin regions (repressed genes) bind to lamins and are localized at the nuclear periphery surrounding nucleoli, while euchromatin (regions of active transcription) are at the center. Therefore, alterations in lamin impact chromatin organization and in turn, gene transcription. Indeed, studies on fibroblasts with genetic inactivation in lamin A, which was previously observed in the nucleoplasm with unknown function, revealed that it contributed to organize *topologically associated chromatin domains* (TADs) (Bronshtein et al., 2015). These findings demonstrated that in addition to their structural roles, lamins also have chromatin organizational functions in the nucleus (Bronshtein et al., 2015). In contrast to these observations, Amendola and colleagues reported that loss of lamin in mouse embryonic stem cells (mESCs) did not alter LADs suggesting that lamin organization of chromatin might be cell-specific or developmentally constrained (Amendola and Steensel, 2015). In fact, subsequent studies showed that in mESCs loss of lamin led to detachment and de-condensation of specific, and not all LADs (Zheng et al., 2018). Furthermore, in these cells, lamin loss did not alter TAD organization, but rather affected TAD-TAD interactions which then resulted in changes in gene transcription (Zheng et al., 2018).

It is becoming progressively clear that lamin-chromatin interactions are not equal in all cell types (Amendola and Steensel, 2015; Zheng et al., 2018). Lamin isoforms contribute differently to nuclear mechanics, a fact that overlays specificity and complexity on how this continuum of interactions affect force sensing and responses in distinct cell types. Work by Vahabikashi and colleagues has extensively characterized the contributions of each lamin isoform in modulating nuclear shape and stiffness using mouse fibroblasts (Vahabikashi et al., 2022). The authors also confirmed the presence of A-type lamin interactions with the actin and vimentin networks *via* nesprin-2G and nesprin-3, respectively, whereas B-type lamins only interacted with vimentin *via* nesprin-3. ECs and vSMCs express both Lamin A/C and Lamin B1 isoforms, thought at different levels (Kim et al., 2018a). In the mouse aorta, vSMCs in the media and fibroblasts in the adventitia express similar levels of prelamin A transcripts (which yields lamin A/C). In contrast, lamin B1 expression is higher in the adventitia and in intimal ECs than in the media (Kim et al., 2018a).

Due to the highly interactive molecular connectivity between lamins, cytoskeletal and LINC proteins, cells with low or depleted Lamin A/C also exhibit changes in LINC complex organization and protein expression, which contribute to defects in nuclear mechanotransduction in laminopathies (Libotte et al., 2005; Hale et al., 2008). Studies using lamin null mice report impaired localization of emerin and nesprin-2, -3 at the nuclear envelope but in a cell type-specific manner (Libotte et al., 2005; Hale et al., 2008). It is important to highlight that in addition to the structural role provided by lamins and LINC complexes, histone modifications, per se, can affect nuclear rigidity and morphology in a manner that is independent of lamins (Stephens et al., 2018).

## 7 PATHOLOGICAL CONSEQUENCES OF IMPAIRED LINKER OF NUCLEOSKELETON AND CYTOSKELETON INTEGRITY

Laminopathies, a group of pathologies caused by mutations in the gene encoding Lamin A/C (*LMNA*), are characterized by rapid aging, higher incidence of atherosclerosis, and premature death caused by cardiovascular dysfunction including heart attack and strokes (Hennekam, 2006). Mutations in *LMNA* produces an altered form of pre-lamin A termed progerin, that is unable to undergo post-translational modifications necessary for the protein to reach its mature form resulting in the accumulation of farnesylated pre-lamin A at the nuclear periphery. This accumulation leads to misshaped nuclei and increased nuclear stiffness (Verstraeten et al., 2008). Hutchinson-Gilford Progeria Syndrome (HGPS) is the most well-studied laminopathy and it primarily affects vascular smooth muscle cells in large blood vessels. Under cyclic strain, progerin accumulation in vSMCs promotes nuclear rupture resulting in progressive cell loss with physiological consequences to blood vessels (Kim et al., 2018a; Kim et al., 2021). Although ECs were believed to be more protected from nuclear ruptures due to their high expression of lamin B1, recent studies suggest that progerin accumulation also negatively affects EC function. Specifically, Danielsson and colleagues recently demonstrated impaired responses to flow including increased cell detachment, dysmorphic nuclei, and micronuclei formation (Danielsson et al., 2021). Interestingly, analysis of aged mice expressing human mutant *LMNA* uncovered an age-dependent decrease in mechanotransduction proteins including vimentin, suggesting that loss of these proteins could potentially contribute to cardiovascular dysfunction in HGPS (Song et al., 2014).

Aged cells taken from otherwise healthy patients also display abnormal nuclear shapes similar to those seen in HGPS patients (Lans and Hoeijmakers, 2006; Scaffidi and Misteli, 2006). Aging is associated with vascular dysfunction (reduced endothelial vasoactive responses) and structural changes of large vessels (Paneni et al., 2017). Several studies in different animal models further support the notion that shear stress profiles also change with age (Bond et al., 2011; Tian et al., 2022). How age-induced changes in flow and nuclear shape affect LINC complex-based mechanosensing and mechanotransdcution is an important avenue for future exploration.

The large number of disorders that result from mutations in LINC proteins highlight the critical role of cytoskeletal-nuclear connections in chromatin organization, cellular resilience and responses to physical forces. Specifically, mutations in both lamin A (*LMNA*) and emerin (*EMD*) lead to Emery-Dreifuss muscular dystrophy (EDMD), utosomal dominant EDMD in *LMNA* mutations and X-linked EDMD in *EMD* mutations (Holaska, 2008; Ishikawa et al., 2020), both associated with cardiac arrythmia (Boriani et al., 2003). Furthermore, mutations in nesprin-1 and -2 were also found to cause EDMD (Zhang et al., 2007). Buschke-Ollendorff syndrome, a rare disorder of increased bone density is caused by a loss-of-function mutation in the inner nuclear membrane protein, Man1 (also known as LEMD3). Man1 antagonizes TGFβ/BMP signaling, though this mutation does not seem to affect mortality in carrier individuals. A recently identified missense mutation in vimentin resulted in accelerated aging similar to Hutchinson-Gilford Progeria Syndrome including alopecia, lipodystrophy, early onset deafness and stroke (Cogné et al., 2020). This mutation at leucine 387 was found to alter the stability of vimentin filaments with increased filament turnover (Cogné et al., 2020). Interestingly, recent studies have identified the presence of keratins, specifically keratin-17 in the nucleus of HeLa cells where it regulates nuclear shape and chromatin organization (Jacob et al., 2020). In cardiomyocytes, desmin is the intermediate filament responsible for maintaining cell and nuclear integrity. Mutations in desmin lead to cardiac dysfunction, including left ventricular noncompaction cardiomyopathy (Brodehl et al., 2018). Altered LINC integrity by deletion of SUN2 was also found to cause age-related cardiac hypertrophy (Stewart et al., 2019). These studies demonstrate that alterations in or loss of proteins maintaining nuclear structure compromises the integrity of force-bearing cells.

## 8 Future Perspectives

Until recently mechanobiology studies in vascular cells have been limited to the identification of sensors and signaling events that induce endothelial responses to shear stress and global transcriptional changes. Recent advances in sequencing technologies have now facilitated the exploration of mechanotransduction pathways at the single cell level, and thus, we anticipate a flurry of studies aimed at gaining deeper information on how distinct physical perturbations change the transcriptional landscape of vascular cells over time. In addition, studies that focus at identifying post-translational modifications as potential functional switches in response to flow will likely bring clarity to the initial triggers that initiate flow responses. Furthermore, a deeper understanding of the components that bridge “integrins to nucleus” and how flow alters the 3D architecture of chromatin are likely to emerge from these efforts. Recently, Sáinz-Jaspeado and colleagues highlighted the role of Palmdelphin, a cytoskeletal interacting protein, in maintaining EC nuclear resilience to physical distress (Sáinz-Jaspeado et al., 2021). Palmdelphin depletion leads to defects in nuclear orientation, nucleoplasmic shuttling, and loss of the nuclear actin cap. Single-nucleotide polymorphisms in palmdelphin can result in calcific aortic valve stenosis which also display impaired nuclear orientation. Though these findings did not appear to be caused by flow or affect nuclear lamins, the defects in nuclear orientation and nucleocytoplasmic shuttling could make ECs more susceptible to disease.

Whether specific nesprin-SUN isoform interactions have different functions in endothelial cells remains unanswered. Work by Buglak and colleagues as a pre-print has implicated SUN1 in vascular junctional integrity *via* its interactions with microtubules (Buglak et al., 2021). SUN1 knock-down ECs also displayed increased contractile function, though the effects on junctional integrity were not dependent on actin. Thus, loss of nuclear membrane proteins can alter mechanoresponses in vascular cells independent of actin dynamics.

While several of the molecular players that integrate the nucleus with the cytoskeleton (LINC proteins) have been characterized, a concrete understanding of their unique functions in the context of vascular mechanotransduction is only at its infancy. How do LINC and intranuclear cytoskeletal proteins impact chromatin modifications and gene expression in response to physical forces is a broad and exciting area of research for years to come.
